# Immuno-metabolic crosstalk in osteoarthritis: from mechanisms to therapies

**DOI:** 10.3389/fimmu.2026.1946261

**Published:** 2026-10-01

**Authors:** Yangfei Yi, Ke Ning, Yitong Ding, Fen Yang, Yufei Li, Yi Zhuo

**Affiliations:** 1 School of Basic Medical Sciences Health Science Center Hunan Normal University, Changsha, China; 2 School of Basic Medical Sciences/School of Nursing, Health Science Center, Hunan Normal University, Changsha, China

**Keywords:** immuno-metabolic crosstalk, macrophages, mesenchymal stem cells, metabolic intervention, osteoarthritis, personalized medicine

## Abstract

**Background:**

Osteoarthritis (OA) is a heterogeneous whole-joint disorder influenced by mechanical stress, aging, previous trauma, genetic susceptibility, subchonrral bone alterations, and localized inflamation.Emerging evidence suggests that immune and metabolic processes alterations may be involved in OA pathogenesis particularly in inflammatory and metabolically dysregulated subtypes.

**Content:**

This narrative review summarizes the bidirectional crosstalk between immune and metabolic processes during OA progression. Human observational studies and experimental models suggest that immune-cell activation and metabolic dysfunction may interact to influence synovial inflammation and cartilage homeostasis although their causal relevance and relative contributions vary across OA phenotypes. We further discuss emerging immunometabolic strategies targeting glycolytic and lipid-metabolic pathways as well as predominantly experimental approaches involving gene editing synthetic biology and stem cell-based therapies.

**Conclusion:**

Integrating multi-omics technologies with personalized medicine may improve patient stratification and dynamic monitoring and could facilitate the future development of more personalized and potentially disease-modifying strategies for OA.Future research should focus on clarifying the dynamic immunometabolic network underlying OA and promoting the clinical translation of innovative strategies through artificial intelligence and interdisciplinary collaboration.

## 1 Introduction

Osteoarthritis (OA), a prevalent chronic condition currently impacting more than 500 million people worldwide is increasingly recognized as a heterogeneous whole-joint disease rather than a single entity driven by a uniform pathogenic mechanism (1, 2). The high prevalence of OA not only has a severe impact on the individual’s quality of life but also places a substantial burden on public health systems (3). OA is predominantly characterized by joint pain stiffness and functional limitations (4). As the disease advances patients frequently encounter a substantial reduction in their daily living and work capabilities, and prolonged suffering may also play a role in the development of psychological disorder (5). Current management strategies for OA primarily include pharmacological therapy, physical therapy, lifestyle interventions, and surgical treatment (6). Pharmacological management mainly relies on non-steroidal anti-inflammatory drugs (NSAIDs) to alleviate pain and improve symptoms (7, 8). However, long-term use of these agents may be associated with gastrointestinal adverse effects and renal impairment (9). Despite substantial efforts to develop disease-modifying osteoarthritis drugs (DMOADs) (10), no generally accepted and approved pharmacological therapy has yet been demonstrated to reliably prevent or reverse OA progression (11) and most candidate DMOADs remain under clinical investigation. Physical therapy and exercise are also important components of OA management (11–13), although their effectiveness may be influenced by healthcare accessibility and patient adherence. In addition persistent joint pain and stiffness can restrict voluntary physical activity potentially contributing to further disease progression (14, 15).

These therapeutic limitations partly reflect the marked biological heterogeneity of OA.Historically osteoarthritis (OA) was largely considered a progressive cartilage wear disease resulting from mechanical overloading (16). Although biomechanical stress remains a major pathogenic factorthis model alone cannot fully account for the heterogeneity of OA. OA is now recognized as a complex whole-joint disease in which mechanical loading ageing previous injury genetic susceptibility local inflammation, and metabolic abnormalities contribute to varying degrees across different disease phenotypes. Accordingly, OA can be broadly categorized into mechanically driven OA post-traumatic OA age-related OA and metabolic OA. Mechanically driven OA is predominantly associated with elevated joint loading and biomechanical stress; post-traumatic OA develops following joint injury and is characterized by persistent inflammation; age-related OA is closely linked to cellular senescence mitochondrial dysfunction impaired tissue repair and age-associated low-grade inflammation; and metabolic OA is frequently associated with obesity insulin resistance dyslipidaemia adipokine imbalance and chronic systemic low-grade inflammation (17). These phenotypes are not mutually exclusive and may coexist or converge on shared downstream processes including synovial inflammation cartilage matrix degradation and subchondral bone remodelling. Therefore, within this heterogeneous framework immunometabolic dysregulation should be considered an important pathogenic dimension or endotypeparticularly in inflammatory and metabolically dysregulated OA phenotypes, rather than a universal mechanism underlying all forms of OA (18).

Local inflammation is a common feature of OA whereas systemic low-grade inflammation is more evident in selected metabolic and inflammatory phenotypes (19). The joint cavity contains a substantial quantity of immune cells. The activation of these immune cells is closely associated with chronic inflammatory responses and may result in further damage to chondrocytes and other articular cells (20). An increase in pro‐inflammatory (M1) polarization within the synovium which is characterized by the elevated secretion of pro-inflammatory cytokines such as tumor necrosis factor‑α (TNF‑α) and interleukin‑1β (IL‑1β) not only intensifies local inflammatory responses in the synovial membrane but also disturbs articular cartilage metabolism resulting in heightened degradation and reduced regenerative capacity (21). Recent studies have revealed a marked imbalance in energy metabolism with osteoarthritis particularly within chondrocytes where dysregulated glycolysis and oxidative phosphorylation contribute to chondrocytes dysfunction. Metabolic dysregulation plays a significant role in the pathogenesis and progression of OA (22). This metabolic shift not only impairs the biosynthetic capacity of cartilage but also has the potential to trigger chronic inflammation by modulating the function of immune cells. In OA the levels of pro-inflammatory cytokines such as IL-6 and TNF-α are increased and metabolic signaling pathways including AMPK mTOR and PPARγ are intricately linked to OA pathology These pathways establish a complex regulatory network. IL-6 and TNF-α not only serve as key players in inflammation but also impact cellular metabolism by suppressing AMPK activity and enhancing mTOR activation thus promoting OA progression (23). Considering the multifactorial characteristics of OA pathogenesis single-target therapies have generally been ineffective (24). Against this backdrop immunometabolic modulation has emerged as a promising therapeutic approach. Recent reviews have examined the links between metabolism and inflammation in OA from different perspectives. Xia et al. focused on metabolic reprogramming and immunometabolic regulation in the context of ferroptosis (1). Liu et al. reviewed inflammation–metabolism crosstalk and emerging therapeutic strategies more broadly (25). Our review builds on this work by examining how immunometabolic dysregulation relates to distinct OA phenotypes and affects the joint as a whole. We do not assume that these processes contribute equally to all forms of OA.We discuss interactions among immune cells chondrocytes synovial fibroblasts and other joint-resident cells alongside the influence of obesity insulin resistance ageing and adipose tissue dysfunction. Where relevant we distinguish findings from OA studies from mechanistic insights obtained in other experimental or disease settings noting the limits of applying these findings to OA. We also assess emerging therapies according to their stage of clinical development and the evidence supporting their use. Particular attention is given to whether promising preclinical findings are supported by human studiesespecially randomized trials. Through this approach we aim to clarify how immunometabolic mechanisms may differ across OA phenotypes and where the evidence remains insufficient to guide treatment.

## 2 Literature search and selection

This narrative review examines immunometabolic mechanisms and related therapeutic strategies in osteoarthritis (OA). We searched PubMed, Embase, and Web of Science from database inception to March 2026 and updated the searches in July 2026. No language restrictions were applied. Search terms covered five areas: OA, immunometabolism, immune and joint-resident cells, metabolic pathways, and therapeutic interventions. These included “osteoarthritis”, “osteoarthrosis”, “immunometabolism”, “metabolic reprogramming”, “macrophage”, “chondrocyte”, “synoviocyte”, “glycolysis”, “fatty acid oxidation”, “mitochondrial dysfunction”, “oxidative phosphorylation”, “AMPK”, and “mTOR.” Treatment-related terms included “metformin”, “gene editing”, “CRISPR”, “stem cells” and “exosomes.” Records from the three databases were combined and deduplicated before being reviewed by three authors. Publications were considered relevant if they addressed immune–metabolic interactions in OA or interventions targeting these processes. The review included human studies, OA-related animal experiments, *in vitro* and ex vivo studies, and relevant reviews. Evidence from other diseases was used only to explain background mechanisms and was distinguished from findings obtained directly in OA.

Studies were prioritized according to the question being addressed. Human studies informed discussions of clinical associations and treatment outcomes, while experimental studies informed the assessment of mechanisms. Findings were interpreted with reference to the study population or model, design, and outcomes, with attention to negative results and differences between studies. The evidence was synthesized narratively, distinguishing clinical observations, experimental findings, and proposed mechanisms. This was not a systematic or scoping review, and some relevant publications may have been missed.

## 3 The immunological characteristics of OA

For a considerable period OA has been recognized as a degenerative disorder mainly propelled by aging and mechanical stress which is characterized by the progressive deterioration of articular cartilage (26). Nevertheless in this process inflammation is not merely a secondary change in the advanced stages but rather plays a critical role in both disease initiation and sustained progression (27). The joint cavity contains a substantial population of immune cells primarily including neutrophils monocytes macrophages B cells T cells NK cells and dendritic cells (28). These cells not only participate in local immune responses but also significantly contribute to the pathological alterations within the joint (29).

### 3.1 Immunopathological alterations in OA

The synovium is an important site of local inflammatory and immune responses during osteoarthritis progression (30). Histologically the synovium consists of an intimal lining layer and a sublining layer both of which may undergo structural remodeling in OA. OA-associated synovitis is characterized by varying degrees of synovial lining hyperplasia stromal fibrosis (31), vascular remodeling and inflammatory cell infiltration (28)^28^. Increased expression of pro-angiogenic mediators particularly vascular endothelial growth factor (VEGF) may promote neovascularization within the synovium (32). Synovitis is not merely a common pathological characteristic of OA but also a vital driver of cartilage degradation pain and disease progression (33). Researchers have identified a population of osteoclast-like cells in the synovium and interstitial fibrous layers. These cells which are characterized by the expression of RANKCTSK and MMP-9 are likely to be involved in joint destruction (34). Traditionally activated macrophages have been broadly classified into pro-inflammatory M1 and anti-inflammatory or tissue-reparative M2 phenotypes. Although the M1/M2 framework remains useful for describing macrophage responses in simplified *in vitro* systems and many preclinical OA studies, accumulating evidence indicates that this binary classification does not adequately capture the diversity of macrophage states present in human OA joints (35). The strength of evidence also varies across experimental settings. Mechanistic studies using cultured macrophages and animal models of OA suggest that macrophages with M1-like inflammatory features can enhance the production of TNF-α IL-1β and cartilage-catabolic mediators whereas M2-like or pro-resolving macrophage states may facilitate inflammatory resolution and tissue repair (36). However these experimentally induced polarization states should not be directly equated with discrete macrophage subsets in human OA synovium. Instead transcript omic and single-cell studies of human OA tissues have revealed diverse macrophage programs associated with inflammation phagocytosis tissue remodeling lipid metabolism and homeostatic functions (37). Importantly comparative analyses of synovial tissues from OA and rheumatoid arthritis (RA) indicate disease-specific differences in macrophage distribution and activation states (38). RA is generally characterized by more pronounced inflammatory macrophage activation whereas macrophages in OA display greater heterogeneity and mixed inflammatory–remodeling featuresr (39). Regulating pro-inflammatory macrophages can alleviate inflammation and mitigate the degradation of cartilage matrix. TRPV1a cation channel associated with pain perception and inflammation has been demonstrated to reduce the polarization of pro-inflammatory, thereby ameliorating OA (40). Through the restoration of mitochondrial membrane potentialthe elevation of glutathione (GSH) levelsand the promotion of intracellular autophagy, pro-inflammatory were effectively repolarized to an anti-inflammatory. This repolarization augmented chondrocyte proliferation and viability concurrently suppressing apoptosis (41). In the early stage of OA the subchondral bone plate experiences thinning along with an increase in porosity which is accompanied by heightened bone resorption activity. Osteoclasts facilitate angiogenesis and nerve ingrowth through the release of factors such as TGF‑β1 and H^+^. Simultaneously they activate immune cells including pro-inflammatory, thus establishing a pro‑inflammatory microenvironment (42). Concurrently osteocytes increase the RANKL/OPG ratio and osteoblasts secrete mediators such as IL‑6 and PGE2, which further promotes osteoclast differentiation and sustains the inflammatory cycle. Immune cells and stromal cells establish a complex network mediated by metabolites and cytokines within which the osteoclast-chondrocyte crosstalk serves as a crucial pathological link. Osteoclasts facilitate chondrocyte hypertrophy and apoptosis through signaling via exosomes and other pathways. Meanwhile chondrocytes recruit and activate osteoclasts by releasing factors such as CXCL12 and HMGB1, thereby establishing a vicious cycle (43). The acidic microenvironment the release of damage‑associated molecular patterns (DAMPs). and the upregulation of nerve growth factor (NGF) further exacerbate pain and immunometabolic dysregulation (44). Therefore the immune microenvironment in OA is characterized by a complex and interrelated state of imbalance which serves as the core pathological basis for driving disease progression and pain.

### 3.2 Key immune cells and inflammatory mediators

T and B cells have been identified in OA synovial tissue with their abundance and activation profiles varying across patients and disease stages (45). Studies of human OA joints have reported changes in CD4^+^ and CD8^+^ T-cell populations including Th1Th17and regulatory T (Treg) cells (46). Th1- and Th17-associated cytokines may contribute to local inflammatory signaling whereas Treg cells may limit inflammation through mediators such as IL-10 and TGF-β (47). B-cell infiltration and occasional lymphoid aggregates have also been observed in subsets of OA synovial samples although their contribution to disease progression remains uncertain. These findings suggest adaptive immune involvement in selected OA phenotypes rather than establishing a universal antigen-driven or immune-complex-mediated disease mechanism. During the recruitment of inflammatory cells neutrophils release pro‑inflammatory cytokines such as IL‑1β and IL‑6generate reactive oxygen species and engage in the activation of matrix‑degrading enzymes, which collectively contribute to cartilage destruction (48) stimulated chondrocytes produce MMPs, TNF-α, IL-6 and IL-8 leading to the degradation of the extracellular matrix (49). Inflammatory stimuli activate nerve endings which leads to joint pain (50). Meanwhile neovascularization and cellular infiltration continuously sustain the inflammatory process thereby establishing a vicious cycle (51). TNF-α and IL-1β are among the most extensively studied inflammatory mediators in OA. Elevated levels of these cytokines have been detected in the synovial tissue and synovial fluid of subsets of patients with OA. Experimental studies indicate that TNF-α and IL-1β can enhance inflammatory signaling and promote catabolic responses in chondrocytes by increasing matrix-degrading enzymes such as MMPs while suppressing extracellular matrix synthesis. However their concentrations and biological effects vary across patients disease stages and experimental settings and neither cytokine alone is sufficient to account for the heterogeneous pathogenesis of OA. (**Figure 1**).

**Figure 1:**
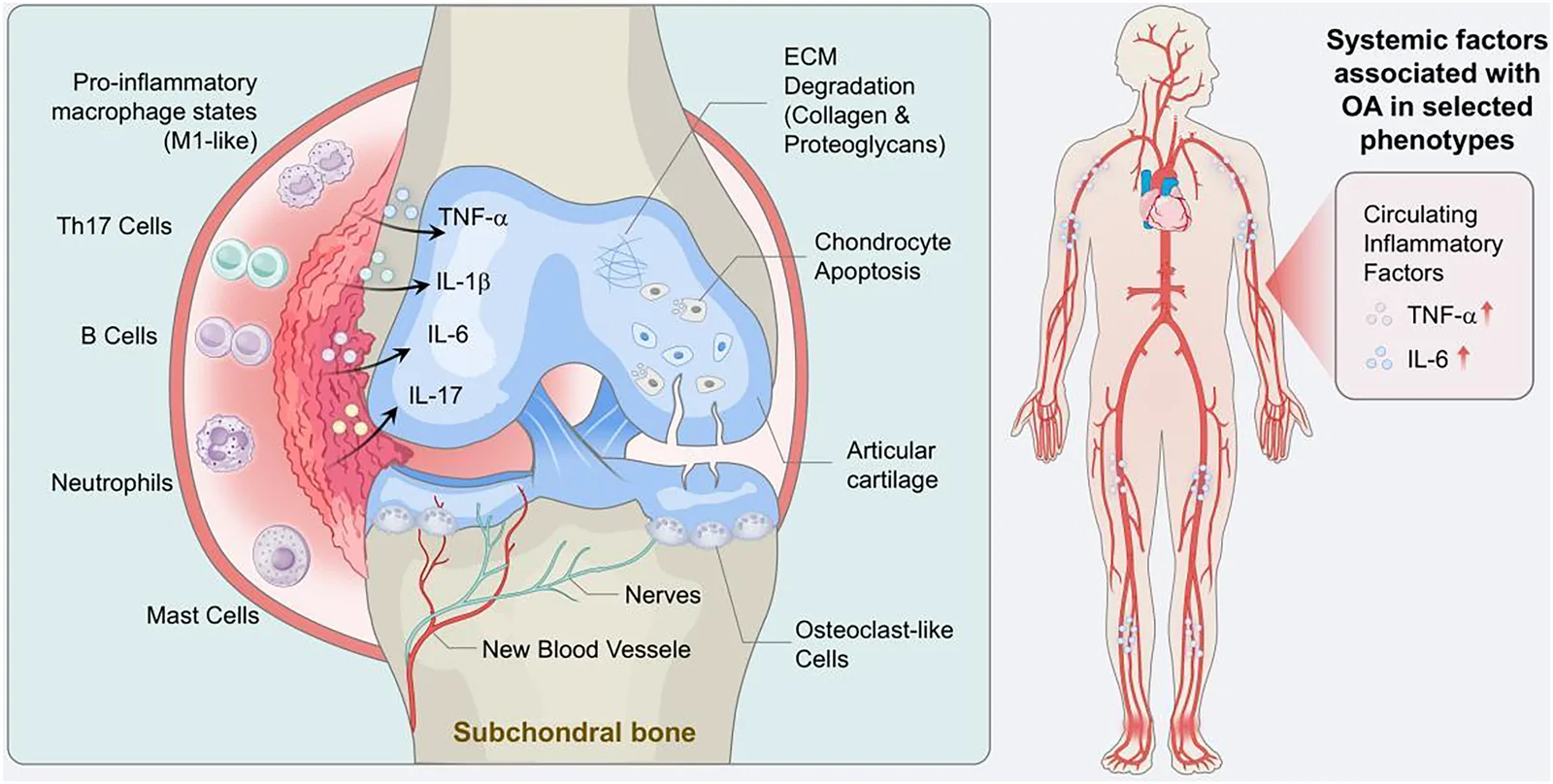
Local tissue alterations and immune features in osteoarthritis and their potential associations with systemic inflammatory factors. The left panel illustrates selected features of the local osteoarthritis (OA) joint microenvironmen tincluding synovial inflammation and vascular remodeling in filtration of immune-cell populations cartilage extracellular matrix degradation chondrocyte apoptosis subchondral bone remodeling and neurovascular changes (26, 28). Pro-inflammatory macrophage states and adaptive immune-cell populations including Th17 cells may contribute to local inflammatory and catabolic signaling; howeve rtheir abundance activation states and functional relevance vary across patients disease stages and OA phenotypes (31). The terms “M1-like” are used here only as operational descriptors of experimentally defined macrophage states and should not be interpreted as fixed or mutually exclusive macrophage identities in human OA (35–37). The right panel illustrates reported associations between selected OA phenotypes and circulating inflammatory mediators including TNF-α and IL-6. Such systemic inflammatory associations are particularly relevant to inflammatory and metabolically dysregulated OA phenotypes and are heterogeneous across patients (17, 23, 26).

## 4 Metabolic characteristics of OA

Based on the immunopathological microenvironment of osteoarthritisthis review expounds upon the intricate network of immune cell infiltration and activation during the progression of OA and their detrimental impacts on joint tissues. Nevertheless the energy sources and initiating signals that drive and maintain this persistent, low‑grade chronic inflammation remain ambiguous. Conventionally, obesitymetabolic syndrome and related conditions are frequently regarded as comorbidities or risk factors for OA. The metabolic characteristics of OA have attracted growing attention in recent research (19). Metabolic factors exert a substantial influence on its pathogenesis especially in the metabolic changes noted in chondrocytes and synovial cells (52).

### 4.1 Mitochondrial dysfunction

As the energy and regulatory hub of eukaryotic cells mitochondria not only regulate cellular energy metabolism but also participate in crucial biological processes such as thermogenesis apoptosis regulation autophagy and cell differentiation (53). Mitochondria play an essential role in maintaining chondrocyte homeostasis. The mitochondrial metabolic dysfunction in OA forms a self-reinforcing vicious cycle that is driven by dysregulation across multiple levels of regulatory networks (54). Under the adverse microenvironment of OA joints which is characterized by hypoxia nutrient deficiency and infiltration of inflammatory cytokines the energy balance of chondrocytes is disrupted. The activity of AMP-activated protein kinase (AMPK)a central energy sensoris diminished thereby impairing its ability to effectively sense and respond to energy stress (55). Concurrently the activity of its essential regulator SIRT1 is also diminished. Cells forfeit their capacity to handle stress and initiate protective metabolic reprogramming. The down-regulation of the AMPK/SIRT1 axis directly results in a substantial suppression of the expression and activity of its key downstream target PGC-1α (16). When the synthesis of nucleus‑encoded mitochondrial proteins is impaired PGC‑1α is unable to effectively activate its downstream transcription factor NRF1which results in a reduction in the production of nucleus‑encoded mitochondrial respiratory chain complex proteins and other essential components (22). A decrease in the expression of the PGC‑1α‑regulated mitochondrial transcription factor TFAM significantly impairs mtDNA replication transcription and repair capabilities leading to a reduction in mtDNA copy number and the accumulation of mutations (56). Owing to the inadequate synthesis of respiratory chain proteins and defects in components encoded by mtDNA the efficiency of the mitochondrial electron transport chain is notably reduced and the impaired oxidative phosphorylation results in a decrease in ATP production (57). Simultaneously electrons are more prone to leak from the damaged respiratory chain where they combine with oxygen to produce an excessive amount of super oxide anions. Given that the defense capacity of SOD2 has already been impaired these reactive oxygen species (ROS) accumulate abnormally within the mitochondria thereby triggering severe oxidative stress (58). Dysfunctional mitochondria that produce excessive ROS ought to be cleared promptly. Nevertheless an excess of ROS not only directly inflicts damage on mtDNA and proteins but also functions as potent signaling molecules thereby activating pro-inflammatory and catabolic pathways such as the NF-κB and MAPK pathways. This in turn further upregulates the expression of MMPs a disintegrin and metalloproteinase with thrombospondin motifs (ADAMTS), and inflammatory cytokines (59). ROS primarily originate from the mitochondrial electron transport chain and the plasma membrane nicotinamide adenine dinucleotide phosphate (NADPH) oxidase system and play a central driving role in the initiation and progression of OA. Under stress conditions excessive mitochondrial ROS production and telomere shortening collectively serve as driving factors for cellular senescence (60). Among these oxidative stress‑induced defects in mitochondrial DNA repair are regarded as a crucial link in the pathogenesis of OA. Excessive ROS not only directly impair mitochondrial structure and function but also induce mtDNA damage and activate the DNA damage response thereby facilitating premature chondrocyte senescence extracellular matrix degradation and subchondral bone loss (61). Due to the reduced activity of mitochondrial complexes II and III in osteoarthritic chondrocytes the production of ROS is further exacerbated (55). ROS inhibits the synthesis of key cartilage matrix components such as glycosaminogly cans and type II collagen by activating signaling pathways like MAPK/ERK. Concurrently it up regulates the expression of type I collagen, MMPs, and various pro-inflammatory cytokines (62). In the initial phases of OAROS can also trigger chondrocyte apoptosis through the PI3K/Akt and caspase pathways. Superoxide dismutase (SOD)a crucial antioxidant enzymepre serves redox homeostasis by catalyzing the transformation of superoxide anions into hydrogen peroxide and oxygen. Glutathione is synthesized within cells to regulate the intracellular redox environment (63). α‑Ketoglutarate (α‑KG) serves as a vital physiological metabolite in the mitochondrial tricarboxylic acid (TCA) cycle and it exhibits diverse functions such as anti‑inflammatory and antioxidant properties. The serum levels of α‑KG decrease with age (64). Thus mitochondrial metabolic dysfunction in OA is not simply a single-step metabolic abnormality. Instead it establishes a self-perpetuating vicious cycle: mitochondrial dysfunction and oxidative stress result in an increased secretion of inflammatory cytokines which intensifies the inflammation and further inhibits mitochondrial biogenesis and repair (**Table 1**).

**Table 1:** Expression and pathological roles of mitochondrial dysfunction-related regulators in OA.

Key genes/TFs in mitochondrial dysfunction processes	Expression changes during OA progression	Pathological changes during OA progression	Reference
AMPK (AMP-activated protein kinase)	Decreased Activity/Downregulated Expression – Diminished response to inflammation and energy stress.	Functional inhibition of AMPK prevents effective transmission of energy crisis signal simpairs protective responses such as mitochondrial biogenesis and autophagy and compromises chondrocyte adaptation to the hostile OA microenvironment thereby exacerbating metabolic dysfunction and catabolism.	(55)
PGC-1α (Peroxisome proliferator-activated receptor γ co-activator 1α)	Significantly downregulated expression – Its transcriptional activity is suppressed.	Reduced expression directly impairs mitochondrial biogenesis leading to an insufficient number of newly generated functionally competent mitochondria to replace or repair damaged ones. This serves as a key molecular basis for the sustained decline in mitochondrial function and the disruption of energy metabolism.	(56)
SIRT1 (NAD-dependent protein deacetylase sirtuin-1)	Reduced Activity/Expression – Diminished regulatory effect on inflammatory stimuli.	Decreased SIRT1 activity leads to diminished activation of PGC-1αwhile its anti-inflammatory and antioxidant protective functions are compromised. This exacerbates inflammatory responses and oxidative stress and impairs the mitochondrial quality control system.	(56)
NRF1 (Nuclear respiratory factor 1)	Due to downregulation of upstream PGC-1αthe activity of its mediated mitochondrial protein transcriptional network is reduced.	As a downstream effector of PGC-1αits impaired function directly reduces the synthesis of core functional proteins such as those in the mitochondrial respiratory chainexacerbating electron transport chain dysfunction and diminishing oxidative phosphorylation efficiency.	(56)
TFAM (Transcription factor Amitochondrial)	Reduced expression associated with mtDNA damage and decreased copy number.	Decreased TFAM expression exacerbates the accumulation of mtDNA damage and insufficient repairleading to impaired synthesis of mitochondrially encoded respiratory chain proteins. This constitutes a key cause of primary and persistent mitochondrial dysfunction.	(56)
SOD2 (Superoxide dismutase 2)	Downregulated expression – Its activity or stability may be impaired.	The diminished defense capacity results in ineffective clearance of mitochondria-derived superoxide anions significantly exacerbating oxidative stress both within mitochondria and at the cellular level thereby rendering mtDNA and mitochondrial proteins more vulnerable to damage.	(58)
mTOR (Mechanistic target of rapamycin)	In OA chondrocytesthis signaling pathway may exhibit aberrant activation particularly under specific pathological conditions.	Overactive mTOR signaling suppresses protective autophagy and mitophagy hindering the clearance of dysfunctional mitochondria. This leads to the accumulation of damaged mitochondria which continuously generate ROS and release pro-apoptotic factors.	(23)

### 4.2 Abnormal glucose metabolism

Articular cartilage is avascular and receives nutrients primarily through diffusion from synovial fluid (65). Chondrocytes therefore reside in a physiologically hypoxic environment and rely heavily on glycolysis for ATP generation while retaining mitochondrial oxidative phosphorylation (OXPHOS) and tricarboxylic acid (TCA) cycle activity (66). Glycolysis is thus an integral component of normal chondrocyte metabolism and increased glycolytic activity should notby itselfbe regarded as pathological. During OA progression inflammatory stimuli and mitochondrial dysfunction can alter the coordination between glycolysis and mitochondrial metabolism. Depending on the cellular and experimental context these changes may involve increased glycolytic fluxreduced oxidative capacitylactate accumulation and disturbed redox homeostasis (67, 68). OA-associated glucose metabolic remodeling involves alterations in glucose transporters and glycolytic enzymes.Ultimately lactate accumulationan acidic microenvironmentand ROS‑mediated oxidative stress are (Glucose transporter 1) GLUT1 intertwined with inflammatory responses. these changes may impair type II collagen and proteoglycan synthesis promote extracellular matrix degradation and compromise chondrocyte survival (69) is a crucial transporter protein that governs the basal glucose uptake in chondrocytes and supplies glucose for glycolysis. It plays a significant role in chondrogenesis, embryonic development and the maturation of the skeletal system. Glucose deprivation increases GLUT1 expression and glucose uptake in both normal and OA chondrocytes (70). Conditional deletion of Slc2a1which encodes GLUT1in postnatal mouse cartilage impairs growth-plate chondrocyte proliferation and matrix production resulting in shortened long bones whereas articular cartilage exhibits reduced cellularity proteogly can lossand progressive fibrosis (16, 71). These findings highlight the requirement for GLUT1-mediated glucose uptake in cartilage homeostasis. Pro-inflammatory cytokines can also increase GLUT1 expression in chondrocytes; however increased expression alone does not establish whether this response is adaptive or contributes to pathological metabolic changes. In experimental temporomandibular joint OA mechanical stimulation activates PIEZO1–STAT1 signaling in MSCs increasing HK2-mediated glycolysis and MIF secretion (72). Beyond chondrocyte-intrinsic mechanisms exosomes derived from inflammatory fibroblast-like synoviocytes enhance HIF-1α-dependent glycolysis in macrophages. Increased HIF-1α occupancy at the promoters of Slc2a1, HK2 is accompanied by elevated expression of GLUT1, PKM2 and LDHA (65), increased glucose consumption and lactate production and M1-like polarization. These changes amplify inflammatory responses and aggravate experimental OA (73). Importantly the functional consequences of individual glycolytic regulators are cell- and context-dependent. For example although PKM2 expression has been reported to be elevated in OA chondrocytes experimental PKM2 suppression reduces glycolytic activity while simultaneously impairing chondrocyte proliferation increasing apoptosis and decreasing COL2A1 and SOX9 expression. These findings suggest that PKM2-dependent glycolysis may contribute to basal chondrocyte metabolic activity and survival under certain conditions and that PKM2 should not be regarded as uniformly pathogenic or protective.FSTL1-induced glycolytic activation in chondrocytes promotes lactate accumulation and H3K18 lactylation which is associated with the induction of fibrotic gene programs. Pharmacological inhibition of the upstream PI3K/mTOR pathway or LDHA reduces H3K18la and fibrotic markers whereas exogenous lactate partially restores this phenotype supporting a functional role for lactate-dependent histone lactylation in this experimental context (74). This clarifies a comprehensive pathogenic pathway in OA ranging from FSTL1-induced glycolytic activation to lactate - mediated histone lactylation and ultimately to the initiation of downstream fibrotic gene programs (73). Harpagide exerts anti-inflammatory effects through the suppression of the glycolytic pathway in response to TNF-α induced inflammation and plays a therapeutic role in the treatment of OA (75). This key glycolytic enzymes enhances glycolytic metabolism facilitates M1 polarization and amplifies the secretion of inflammatory cytokines that cause damage to cartilage thus exacerbating OA (76).

### 4.3 Other metabolic abnormalities

Previous research has indicated substantial alterations in the plasma levels of palmitic acid γ‑linolenic acid and 3-hydroxyoctadecanoic acid among patients suffering from OA. These abnormal lipid metabolites may engage in and expedite the progression of OA by regulating cellular activity and cytokine secretion. For example stearic acid can trigger the lactate‑HIF-1 signaling pathway in chondrocytes prompting the production of pro‑inflammatory cytokines and consequently aggravating the pathology of OA. Moreover palmitic acid and oleic acid facilitate the generation of ROS via the NADPH oxidase 4 (NOX‑4) signaling pathway eventually resulting in chondrocyte apoptosis (77). Lipids function not only as the fundamental structural constituents of cellular and organelle membranes but also as crucial regulators of cartilage structure and function. Through the maintenance of membrane fluidity participation in signal transduction and facilitation of cellular adaptation to environmental stress lipids play a vital role in cartilage homeostasis (78). Beyond their metabolic functions lipids also act as precursors for a variety of bioactive molecules. For instance there are inflammatory mediators derived from arachidonic acid (AA) and specialized pro‑resolving mediators (SPMs) that actively facilitate the resolution of inflammation. These molecules assume central roles in regulating cartilage inflammation and the repair processes. It has been observed that the dysregulation of lipid metabolism significantly impacts the initiation and progression of OA. Abnormal lipid accumulation within chondrocytes promotes the progression of OA by inducing cellular dysfunction and inflammation (79). A significant dysregulation of lipid metabolism is observed in the cartilage tissues of patients with OAas evidenced by the abnormal accumulation of triacylglycerols (TAGs) and a substantial decrease in docosahexaenoic acid (DHA) levels (80). Amino acids also exert crucial functions in cartilage metabolism. In the context of OA chondrocytes experience significant changes in the uptake and utilization of amino acids. For examplethe metabolism of amino acids like glutamine and arginine may be inhibited (81) thereby compromising chondrocyte proliferation and anabolic capacity. This phenomenon is presumably attributable to the stress response of chondrocytes within an inflammatory environment which disrupts the overall cellular metabolic homeostasis (82) (**Figure 2**).

**Figure 2:**
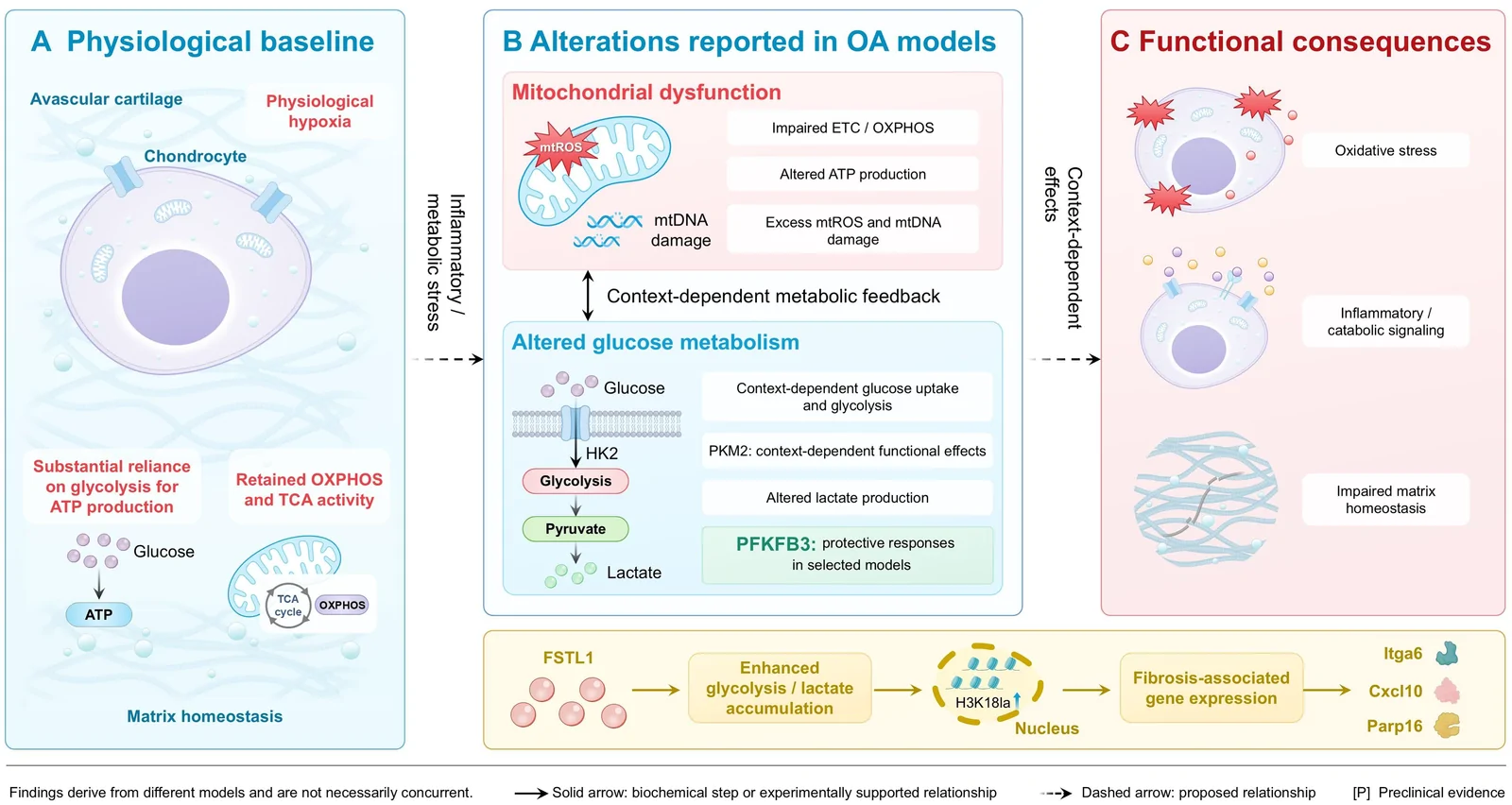
Energy metabolism in healthy chondrocytes and context-dependent metabolic changes in osteoarthritis. **(A)** Articular cartilage is avascular, and chondrocytes normally occupy a relatively hypoxic environment. Glycolysis supplies much of their ATP, although mitochondrial oxidative phosphorylation (OXPHOS) and the tricarboxylic acid (TCA) cycle remain active and help maintain cellular function and extracellular matrix homeostasis (16, 22, 69). **(B)** Studies of osteoarthritis (OA) and related experimental models have identified mitochondrial abnormalities, including impaired electron transport and OXPHOS, altered ATP production, increased mitochondrial reactive oxygen species (mtROS), and mitochondrial DNA (mtDNA) damage (57–59). Changes in glucose uptake, glycolytic flux, and lactate production vary across disease settings and experimental conditions (69–71). HK2 has been implicated in glycolytic remodeling (69, 72), but individual glycolytic regulators can have different effects. PKM2 may help sustain glycolysis and chondrocyte homeostasis in some experimental settings, while PFKFB3-dependent glycolysis has shown protective effects in human OA cartilage and experimental chondrocyte models. **(C)** These metabolic changes may promote oxidative stress, inflammatory and catabolic signaling, and matrix disruption, depending on the biological context (61, 62, 69, 77). The lower panel depicts a pathway described in preclinical OA research: FSTL1 promotes glycolysis and lactate accumulation, followed by increased H3K18 lactylation and expression of fibrosis-associated genes in chondrocytes. Solid arrows denote biochemical reactions or relationships supported by experiments in the cited models; dashed arrows denote proposed relationships or effects that vary with context.

## 5 Immune-metabolic crosstalk in OA

Metabolic dysregulation functions as an active modulator in OA directly engaging in the regulation of the joint immune microenvironment and serving as the material and energetic foundation for the persistence of inflammatory responses. Through the exchange of metabolites and signaling interactions between immune cells and joint-resident cells a vicious cycle of metabolism-immunity is established (83). Reprogramming immune responses through the modulation of metabolic pathways (84) or correcting metabolic imbalances by modulating the immune status to regulate the progression of OA (85), transitioning from symptomatic anti-inflammation to etiological homeostatic restoration (15).

### 5.1 Local immunometabolic mechanisms and their links to systemic risk factors in OA

Within inflammatory and metabolically dysregulated OA contexts metabolic reprogramming of immune cells particularly macrophages may contribute to local inflammation and disease progression (86). Multiple key metabolic pathways including the tricarboxylic acid cycle fatty acid metabolism and amino acid metabolism have been identified (87). In LPS-activated macrophagesPKM2 has been shown to regulate HIF-1α activity glycolytic reprogramming and IL-1β production (88). These findings provide a mechanistic framework for inflammatory macrophage metabolism but were obtained in non-OA experimental systems and do not, by themselves establish the same pathway in OA synovial macrophages (87). The immunometabolic axis represents an emerging therapeutic frontier in OA. Recent work has identified SIK1 as a key regulator bridging macrophage-mediated inflammation and metabolic dysregulation; its agonist AICAR has been demonstrated to reprogram macrophage glucose and lipid metabolism while concomitantly attenuating pro-inflammatory responses.The subsequent development of a liposomal hydrogel system for sustained AICAR delivery further underscores the translational potential of targeting immunometabolic crosstalk in OA therapy (89). Metabolite-mediated interactions between immune and stromal cells have been investigated in inflammatory arthritis although the disease context differs across studies. In collagen-induced arthritis rats and associated synovial fibroblast experiment ssuccinate–HIF-1α–NLRP3 signaling was implicated in IL-1β production and fibroblast activation (90). These findings were obtained in an inflammatory arthritis model rather than an OA model; their relevance to OA therefore represents a mechanistic extrapolation.These results should not be interpreted as direct evidence of the same mechanism in OA synovial fibroblasts.Separate OA-focused experiments have reported protective effects of the itaconate derivative 4-octyl itaconate in IL-1β-stimulated chondrocytes and a mouse OA model involving Nrf2/STING/NF-κB signaling. This provides preclinical evidence for a pharmacological interventionbut does not establish that endogenous macrophage-derived itaconate mediates the same effects in human OA. In an OA-relevant *in vitro* studybasic calcium phosphate crystals increased glycolytic activity and M1-associated markers in human monocyte-derived macrophageswhile 2-deoxyglucose reduced crystal-induced activation (91). These findings support a metabolic contribution to crystal-induced macrophage activation under the tested conditions but do not establish that this pathway drives OA progression *in vivo*.Howeverthe supporting evidence differs between targets: 4-octyl itaconate has been evaluated in preclinical OA models whereas the succinate studies discussed above primarily concern non-OA inflammatory arthritis. Their therapeutic implications for human OA therefore require separate validation.At the tissue level, specific anatomical structures and the local microenvironment integrate and amplify intercellular crosstalk to form sustained pathological circuits (92). In this processthe infrapatellar fat pad (IPFP) serves as a crucial hub functioning not only as a mechanical buffer but also as an active immunometabolic endocrine organ (93). Under conditions of metabolic dysregulation hypertrophied adipocytes alter their secretory profile to a pro-inflammatory phenotype. This alteration is characterized by an increased release of leptin and resistina decreased secretion of adiponectin and the diffusion of free fatty acids and adipokines directly into the adjacent synovial tissue (94). This altered secretory profile may drives macrophage polarization toward the pro-inflammatory M1 phenotype and activates synovial fibroblasts (39). This process establishes two characteristic pathogenic circuits within the joint: the lipid-macrophage-inflammation vicious cyclein which abnormal lipid metabolites activate the TLR4/NLRP3 signaling pathway in macrophages and the activated macrophagesin turnsecrete inflammatory cytokines that further inhibit lipid efflux in chondrocytes resulting in intracellular lipid accumulation; and a lactate-HIF-1α-glycolysis positive feedback loop (95). The hypoxic and inflammatory microenvironment stabilizes HIF-1α expression which upregulates key glycolytic enzymes leading to excessive lactate production. This accumulated lactatein turncan further stabilize HIF-1α (96). Furthermore the chemokine MCP-1which is secreted by activated cells (97) consistently recruits additional immune cells to the joint and facilitates pro‐inflammatory (M1) polarization through receptor signaling. This process works in synergy with the aforementioned metabolic circuits to establish and maintain a long-lasting pro-inflammatory microenvironment (98).

At the systemic level metabolic status ageing adipose-tissue dysfunction insulin resistance (99), and gut microbial alterations may modify the local joint environment and influence OA susceptibility or progression (100). These components not only augment the mechanical load but also exert an influence on the joint by triggering chronic low-grade systemic inflammation. Moreovert he imbalance of adipokines derived from adipose tissue directly modulates joint inflammation (101). Insulin resistance and hyperglycemia lead to the impairment of chondrocyte anabolic function and facilitate the accumulation of advanced glycation end-products. Meanwhile dyslipidemia triggers chondrocytes to generate pro-inflammatory factors and matrix-degrading enzymes through the activation of pathways such as LOX-1 and NF-κB (102). Concurrently the gut microbiota and their metabolites contribute to OA pathogenesis through the gut-joint axis (103) Dysbiosis can result in decreased production of anti-inflammatory short-chain fatty acids and a shift in tryptophan metabolism toward the pro-inflammatory kynurenine pathway (104). Intestinal barrier dysfunction permits lipopolysaccharides (LPS) to enter the bloodstream thereby triggering chronic endotoxemia and perpetuating systemic inflammation. The aging process further disrupts immunometabolic homeostasisat which point inflamm aging intersects with metabolic aging. Senescent cells secrete a senescence - associated secretory phenotype (SASP) that is abundant in factors such as IL-6 and IL-1β. These factors not only directly promote inflammation but also interfere with insulin signaling and lipid metabolism. The synergistic effects of immunosenescence metabolic aging and mitochondrial dysfunction significantly compromise the joint’s ability to maintain intrinsic repair and homeostasis (105). The interaction among these three levels collectively constructs a comprehensive pathological network that drives the onset and progression of OA. Simultaneously it presents crucial targets for the development of novel therapeutic strategies focused on key metabolic nodes to interrupt pathological crosstalk.

### 5.2 The regulation of immune responses by metabolites

In OA glycolysis stands as the fundamental basis of metabolic reprogramming in macrophages. SpecificallyM1-polarized macrophages demonstrate the Warburg effect resulting in a significant accumulation of glycolytic intermediates including pyruvate and lactate. These metabolites act not only as energy substrates but also as signaling molecules that trigger pathways such as HIF-1αthus enhancing the release of pro-inflammatory cytokines (106). Fatty acid metabolism also plays a crucial role in modulating macrophage polarization. Mitochondrial STAT3through driving CPT1a-mediated fatty acid oxidation facilitates M1 polarization in response to LPS stimulation thus enhancing the expression and release of inflammatory cytokines (107). This regulatory function is further associated with choline metabolism. Choline enhances the utilization of fatty acids prevents the abnormal accumulation of lipids and supports the remodeling of macrophage phospholipids and metabolic homeostasis (108). Glutamine metabolism modulates the epigenetic regulation of macrophages via α-ketoglutarate which promotes M2 polarization. This effect is consistent with the regulation mediated by AICAR suggesting that coordinated changes in multiple metabolites jointly influence macrophage polarization. Damage-associated molecular patterns (DAMPs)including S100A8uric acid and calcium oxalate crystals have the potential to activate the NLRP3 inflamma some and intensify local inflammatory responses (109). Succinate enhances IL-1β secretion via its receptor SUCNR1 and promotes M1 polarization whereas itaconate alleviates synovial inflammation by suppressing both glycolysis and oxidative phosphorylation (110). These findings emphasize the therapeutic potential of metabolic interventions. Targeting the pathways associated with glucose or fatty acid metabolismor modulating specific DAMPs may mitigate OA inflammation through the reprogramming of macrophage polarization.

### 5.3 Immunometabolic microcircuits

The increasingly apparent association between individual components of metabolic syndrome and osteoarthritis has led to the identification of a distinct metabolic phenotype in OA (111). These metabolic disturbances in conjunction with low-grade systemic inflammation have been demonstrated to have an adverse impact on multiple joint tissues which contributes to the degeneration of the entire joint in OA (112). Studies suggest that chronic low-grade inflammation mediated by obesity and metabolic syndrome has the potential to trigger and promote the onset and progression of OA through the release of endogenous inflammatory mediators which in turn exacerbate joint inflammation (113). Obesity not only increases mechanical load leading to articular cartilage damage but also more critically acts as an active endocrine organ through adipose tissue which secretes various adipokines such as leptinadiponectin and visfatinas well as pro-inflammatory cytokines (114). This triggers a systemic low-grade inflammatory response that disrupts tissue homeostasis in articular cartilage synovium and subchondral bone thereby accelerating cartilage degradation and joint structural destruction (115). Obesity-associated insulin resistance and hyperglycemia exacerbate oxidative stress accumulation of advanced glycation end-products and mitochondrial dysfunction thereby impairing chondrocyte repair capacity and promoting apoptosis (116). Dyslipidemia also plays a significant role in OA progression as elevated cholesterol and oxidized low-density lipoprotein can activate the LOX-1 receptor and NF-κB signaling pathway, inducing chondrocytes to produce more matrix metalloproteinases and pro-inflammatory factors, thereby disrupting the cartilage matrix (117). Simultaneously, elevated levels of free fatty acids have the potential to activate synovial macrophages thereby exacerbating localized inflammation. Diabetes exerts an influence on joint health via two distinct pathways: chronic hyperglycemia and insulin resistance. A high-glucose environment results in the accumulation of intracellular glucose within chondrocytes which in turn initiates oxidative stress and inflammatory cascades. Meanwhile impaired insulin signaling inhibits the anabolic function of chondrocytes, further undermining the joint’s self-repair capacity (118). Hypertension may indirectly contribute to cartilage degeneration by impairing subchondral bone perfusion which leads to localized ischemia and abnormal bone remodeling (119, 120). The interaction between inflammation and metabolic syndrome forms a cyclical feedback mechanism that drives the progression of OA. On one hand inflammatory responses have the potential to exacerbate the symptoms of metabolic syndrome (121). For example chronic inflammation can undermine insulin sensitivity and disrupt lipid and carbohydrate metabolism thus aggravating obesity and other metabolic irregularities. In contrast, the existence of metabolic syndrome also influences the severity of inflammation (122), as obesity is often linked to a systemic inflammatory state that intensifies OA pathological alterations (123). Metabolic syndrome has the potential to modify the local biological environment within the joint thereby reducing the cartilage’s repair capacity and making it more susceptible to structural damage. Hyperglycemia additionally enhances the formation of advanced glycation end products (AGEs)which have toxic impacts on both chondrocytes and inflammatory cells further facilitating the progression of OA (124).

The interplay among lipids macrophages and inflammation constitutes a fundamental element of the immunometabolic axis in OA. Abnormal lipid species including cholesterol crystals and oxidized low-density lipoprotein function not only as damage signals but also as metabolic danger signals. These signals directly stimulate macrophage polarization towards an M1 phenotype by activating the surface Toll-like receptor 4 (TLR4) or the NLRP3 inflammasome in synovial macrophages (125). This, in turninhibits key genes associated with cholesterol efflux in chondrocytes resulting in secondary intracellular lipid accumulation (126). Lipid accumulation initiates macrophage activation and elevates the release of inflammatory cytokines, establishing a self-perpetuating cycle that exacerbates lipid metabolism, which is a crucial mechanism underlying the pathology of OA (127). Drawing parallels with the concept of metabolic inflammation in atherosclerosis analogous metabolic associations are discernible in the pathogenesis of OA (128). In atherosclerosis, a pernicious cycle is established between abnormal lipid accumulation and inflammatory responses which leads to vascular endothelial dysfunction and arterial stiffening (129). Thus dysregulated lipid metabolism not only promotes heightened inflammatory responses but is further aggravated by the persistence of inflammation itself.

Elevated cholesterol levels facilitate the transfer of mitochondria from bone cells to chondrocytes through intercellular nanotubes. The transferred mitochondria are enriched with the Nudt8 protein which degrades CoA resulting in the release of mtDNA into the cytoplasm. Subsequently this mtDNA activates the cGAS-STING inflammatory pathway thereby triggering chondrocyte inflammation and extracellular matrix degradation (130). This process not only directly initiates cartilage inflammation but also enhances inflammatory signaling through the promotion of intercellular mitochondrial transfer between chondrocytes and regulates synovial macrophage polarization, thereby establishing a self-perpetuating cycle of metabolic-inflammation dysregulation (131). Research has demonstrated that osteocyte mitochondria function as metabolic and inflammatory signal relays establishing a transcellular metabolic-inflammatory communication within avascular cartilage tissue. By targeting this mechanismpantethine exhibits therapeutic potential in OA through restoring CoA levels stabilizing mitochondrial membranes reducing the leakage of mtDNA and consequently suppressing the activation of the cGAS-STING pathway which underscores its promise as a metabolic-inflammatory targeted strategy for OA (54).

Furthermore, alterations in extracellular matrix components induced by metabolic dysregulation have the potential to activate critical signaling pathways including the mitogen-activated protein kinase (MAPK) and phosphatid linositol 3-kinase/protein kinase B (PI3K/Akt) pathways. This activation subsequently influences the phenotype and function of chondrocytes and fibroblastswhich, in turn, leads to an exacerbation of inflammatory responses and matrix degradation (132). Additionally the autophagic process which serves as a core intracellular quality-control systemis often impaired in OA (133). Dysfunction of autophagy results in the ineffective clearance of abnormal protein aggregates and damaged organelles, which exacerbates oxidative stresslipid accumulation and aberrant inflamma some activationthus establishing another pathogenic feedback loop (134).

## 6 Advances in immunometabolic therapeutic strategies for OA

### 6.1 Immunometabolic regulation: a novel paradigm for OA treatment from molecular mechanisms to personalized intervention

Recent research has elucidated the intricate interplay between the immune system and metabolic pathways. Therapeutic strategies that target this crosstalk present novel avenues for the treatment of OA (135). Studies indicate that during the progression of OA metabolic reprogramming in macrophages significantly influences their polarization state and function (136, 137). M1 macrophages enhance glycolysis and fatty acid metabolism to drive the production of inflammatory factors (138). In OA therapytargeting macrophage metabolic pathways for instance by utilizing glycolysis inhibitors (e.g.2 - DG)amino acid metabolism modulators or fatty acid metabolism regulatorscan potentially facilitate the shift from M1 to M2 macrophage polarization. This in turn can alleviate local inflammation and promote cartilage protection (139). In OA endogenous DAMPs such as S100A8 and uric acid play a pivotal role in driving inflammatory responses (140). Modulating DAMP levels or inhibiting their downstream signaling pathways can reduce inflammation within the joints of OA patients (141). The development of drugs targeting DAMP such as antibody-based or small molecule interventions presents a promising new strategy for mitigating immune responses associated with OA (142). In individuals suffering from osteoarthritis dietary adjustments intended to reduce the consumption of pro-inflammatory foods may mitigate clinical symptoms (143). A Mediterranean-type dietary pattern incorporating olive oil and fish may favorably influence selected inflammatory and cartilage degradation biomarkers in OA although its effects on joint pain remain uncertain (144). Further more an increased dietary intake of antioxidant compounds such as vitamins C and Ein conjunction with phytochemicals may also contribute to the alleviation of localized inflammation. Considering the well-established correlation between obesity and the pathogenesis of osteoarthritis weight management continues to be a crucial aspect of care for individuals with this condition (145). Optimizing body mass index (BMI) reduces mechanical joint loading which can substantially decrease systemic and local inflammatory markers relieve pain and decelerate disease progression in OA (146). Metformina well-established glucose-lowering agenthas been demonstrated in preclinical investigations to alleviate osteoarthritis pathology in murine anterior cruciate ligament transection (ACLT) models. It achieves this by remodeling lipid availability and ameliorating chondrocyte ferroptosis via the activation of the AMPK/ACC signaling pathway (147). Collectively emerging evidence indicates that therapeutic strategies designed to modulate the abnormal immune response or rectify metabolic dysregulation constitute a feasible and promising approach for the management of OA (148–152). A comprehensive and individualized intervention strategywhich integrates dietary modification physical activity and pharmacotherapyis frequently required to simultaneously optimize glycemic controllipid profilesand blood pressuret hereby enhancing the overall metabolic environment in patients with OA.

Cytokine-targeted therapies have attracted interest because inflammatory mediators contribute to the pathophysiology of osteoarthritis (OA) (153). These therapies include anakinraan interleukin-1 (IL-1) receptor antagonistand adalimumaba tumor necrosis factor-α (TNF-α) inhibitor. Howeverrandomized placebo-controlled trials have not shown consistent clinical benefits.A multicenter randomized double-blind trial evaluated a single intra-articular injection of anakinra (50 or 150 mg) in patients with symptomatic knee OA (154). Neither dose significantly improved WOMAC pain stiffness or physical function scores compared with placebo. No significant improvements were observed in biomarkers of cartilage turnover. Similarly in patients with refractory hand OA adalimumab was not superior to placebo for pain relief or most secondary clinical outcomes. A subsequent randomized trial in erosive hand OA also found no significant benefit of adalimumab for pain synovitisor bone marrow lesions.Current randomized evidence therefore does not establish the efficacy of anakinra or adalimumab for OA (155). However treatment responses may vary by joint sitein flammatory phenotype and disease stage. Differences in treatment regimens routes of administration and outcome measures may also affect the findings. Further adequately powered trials with phenotype-based stratification are warranted.Given the multi factorial nature of OA unimodal therapeutic approaches typically result in suboptimal outcomes. As a consequence there is an increasing justification for the implementation of combination strategies that incorporate immune modulators metabolic agents and lifestyle interventions. This multi-targeted paradigm which is designed to simultaneously address the immunometabolic crosstalk inherent in osteoarthritis has substantial potential for optimizing treatment efficacy and improving patient quality of life. The current trend in osteoarthritis management is marked by a transition towards comprehensive and personalized care. A more profound understanding of the interaction between the immune system and metabolism is propelling the development of precisely targeted interventions. Future research should therefore focus on validating these mechanisms in human OA defining clinically relevant phenotypes and determining whether immunometabolic targeting can translate into meaningful symptomatic or structural benefits.

### 6.2 Precision medicine in OA: integration of multi-omics for patient stratification and dynamic monitoring

To address the significant heterogeneity in both pathophysiology and clinical manifestations among patients with OA it is of utmost importance to establish a dedicated immunometabolic classification system. This framework will utilize multi-omics technologies for comprehensive clustering analysis integrating multidimensional data from transcriptomic metabolomic proteomic and immuno phenotypic profiling (156). Integrating multiple omics layers may help characterize the molecular heterogeneity of OA and identify biomarkers relevant to disease progression and treatment response. This approach may improve our understanding of OA pathogenesis and inform the development of more personalized diagnostic and therapeutic strategies (157). The comprehensive integration of diverse omics data facilitates the elucidation of inter-patient variations at the cellular and molecular levels thus clarifying distinct etiological and pathological mechanisms (158). The disease process may be primarily sustained by profound metabolic disturbances triggered by persistent low-grade inflammation, or alternatively intensified by a state of heightened adipokine activity (159). This precise stratification facilitates the development of personalized treatment regimens tailored to individual patients, thus enhancing therapeutic efficacy and reducing the likelihood of unnecessary adverse effects (160). Furthermore the rapid progress in wearable technology and liquid biopsy has made it possible to conduct dynamic monitoring of patients with OA. These tools enable the continuous collection of real-time physiological data including heart rateactivity levels and sleep quality, and the periodic assessment of disease activity and treatment response through biomarkers obtained from blood or other biofluids. This continuous surveillance allows for timely therapeutic adjustments according to the patient’s status promoting a more responsive and precise management approach (161).

The establishment of an immunometabolic classification framework for OA in combination with contemporary real-time monitoring technologies allows for a more profound comprehension of the disease’s complexity and promotes the execution of personalized care. This methodology facilitates more efficient and targeted patient assistance ultimately improving the quality of life. Such advancements carry significant theoretical implications and provide valuable guidance for future clinical practice.

### 6.3 Targeted pathway reprogramming for OA therapy

Current research findings and therapeutic methodologies underscore the burgeoning limitations of mono therapy, which targets a single molecular factor, especially in complex pathological conditions such as OA. This approach may be inadequate for effectively tackling the intricate interactions among multiple dysregulated biological pathways (162). Therefore, a conceptual shift is justified towards an integrated therapeutic paradigm that targets the immunometabolic regulatory network and core organelle function (159). Coordinated modulation of key regulatory mediators enables a more holistic and effective intervention in disease progression (163). Core molecular targetssuch as AMPK mTOR (mammalian target of rapamycin)and the NLRP3 inflammasome serve as critical nodes linking metabolic and inflammatory pathways (164). AMPKa central regulator of cellular energy homeostasis facilitates fatty acid oxidation and glucose uptake in low-energy conditions consequently exerting downstream anti-inflammatory effects (165). The mTOR signaling pathway regulates cellular growth proliferation and metabolism in response to environmental signals and its dysregulated activation is significantly involved in the pathogenesis of multiple chronic diseases (166). The NLRP3 inflamma some functions as a crucial sensor of both endogenous and exogenous danger signals. Its over-activation can trigger chronic inflammation and tissue damage thereby rendering the development of specific NLRP3 modulators a promising therapeutic approach for OA. Simultaneously improving mitochondrial function in articular cells including chondrocytes and synoviocytes ought to be prioritized as a supplementary research emphasis (167). Mitochondria play a central role in cellular energy metabolism. Their dysfunction leads to a compromise in ATP production and an amplification of oxidative stress which in turn exacerbates inflammation and tissue injury. Strategies focused on promoting mitochondrial biogenesis restoring functional integrity and enhancing energetic efficiency have the potential to improve the metabolic state of chondrocytes and synoviocytes reduce oxidative damage and ultimately slow down the progression of OA (168).

### 6.4 Advancing immunometabolic therapies for OA through cutting-edge biotechnology

Emerging Biotechnologies in Osteoarthritis: New Horizons for Targeting Immunometabolism.In a surgically induced mouse OA model, intra-articular CRISPR/Cas9-mediated ablation of MMP13 or IL-1β attenuated structural deterioration, whereas NGF ablation alleviated pain but exacerbated joint damage (169). These findings demonstrate target-dependent effects in a preclinical model and do not establish durable clinical efficacymetabolic restorationor reduced medication requirements in patients with OA (170). An OA-focused study developed an MMP-responsive hydrogel containing lipid nanoparticles loaded with engineered circular RNA encoding an NF-κB inhibitor.The platform showed protective effects in an OA rat model and human OA cartilage cultured ex vivo (171). This represents preclinical evidence for an engineered RNA delivery systemrather than an inflammation-sensing engineered-cell therapy. Engineering immune cells to sense and modify the OA joint environment remains a prospective strategy requiring direct OA-specific validation; its efficacy cannot be inferred from the RNA delivery study (172). In recent yearsMesenchymal stromal/stem cell (MSC)-based approaches have been extensively investigated in OA because of their potential paracrine immunomodulatory and tissue-supportive effects (173). Preclinical studies suggest that Culturing stem cells under well-defined redox conditions can enhance their chondrogenic differentiation capacity consequently improving tissue-restorative outcomes in the local microenvironment (174). This integrated strategy fundamentally enhances the cell-intrinsic regenerative potential in OA converting therapeutic cells from passive agents into active participants in the self-repair process (175). But MSC-based interventions should currently be regarded as promising but still investigational and their disease-modifying or durable cartilage-regenerative efficacy has not been conclusively established.The convergence of gene editing and stem cell technology shows potential for the development of smart stem cells that can respond intelligently to inflammatory and diseased microenvironments. These cells can be engineered not only for the targeted release of reparative factors but also for real - time disease monitoring. The integration of gene therapy with cellular treatment significantly enhances therapeutic precision and efficacy. Simultaneously the integration of artificial intelligence (AI) is bringing about a paradigm shift in OA management (176). AI technologies possess the capability to integrate and analyze multimodal data sourced from both clinical and experimental contexts facilitating more precise disease prognostication and the formulation of personalized treatment regimens (177). For instance, through the synthesis of patient-specific genetic profiles, inflammatory biomarkers, and imaging data artificial intelligence can assist in the identification of high-risk individuals and the optimization of the timing of therapeutic interventions (178). These intelligent analytical methods not only improve the specificity of treatment but also facilitate the proactive identification of potential therapeutic failures thus providing valuable decision-making support to clinicians. Significantly, stem cell therapy presents distinct advantages for immunometabolic modulation in OA (179). MSCs possess a dual therapeutic capacity. They are capable of differentiating into chondrocytes and osteocytes thereby directly contributing to the structural repair of joints. Additionally they secrete a variety of growth factors through paracrine signaling to promote tissue regeneration. Simultaneously their potent immunomodulatory functions which are mediated by the regulation of T cellsB cellsand macrophagesfacilitate a transition of the joint microenvironment towards an anti-inflammatory state (180). A crucial mechanism encompasses the polarization of macrophages towards an anti-inflammatory M2 phenotype along with the simultaneous reduction in the levels of pro-inflammatory cytokines such as IL-1β and TNF-α (181). MSCs have the potential to alleviate metabolic disturbances within the OA joint through the enhancement of mitochondrial function and the modulation of glucose/lipid metabolism. Moreover the exosomes secreted by them contain a variety of miRNAs that are involved in the regulation of metabolic pathways (182). Melatonin modulates multiple signaling pathways involved in immunity and metabolism, thereby attenuating inflammation, oxidative stress, and chondrocyte death and potentially slowing the progression of osteoarthritis (183). By simultaneously exerting tissue-reparative immunomodulatory and metabolic regulatory effects MSCs offer a novel comprehensive therapeutic approach for the management of OA (184). Taken together these emerging technologies provide important research platforms for investigating OA immunometabolism but their translational maturity varies substantially. Their therapeutic potential should therefore be interpreted according to the level and context of the supporting evidence rather than extrapolated directly from preclinical efficacy to clinical benefit (**Table 2**).

**Table 2:** Representative immunometabolic interventions in osteoarthritis: evidence from preclinical studies to clinical translation.

Agent	Experimental model or clinical population	Routedoseand duration	Study design and sample size	Principal outcome	Major limitations	Evidence level	Clinical trial/OA regulatory status	Ref
Metformin	IL-1β-treated murine chondrocytes; ACLT-induced OA mice; human OA cartilage (damaged vs. relatively intact); chondrocyte–RAW264.7 chemotaxis coculture.	Metformin: 16.5 mg/mLintra-articular injection every 3 days for 8 weeks.	10-week-old male C57BL/6J mice: shamACLT+vehicleor ACLT+metformin (n=5/group); knee joints collected 8 weeks post-surgery.	Metformin reduced OA cartilage damagesubchondral sclerosisosteophytesand synovitisalongside reduced lipid dysregulation–associated chondrocyte ferroptosis.	Limited by small samples (n=5/group)male-only ACLT miceshort follow-upand no human treatment data.	Preclinical *in vitro* and *in vivo* evidence	Preclinical OA study; no clinical trial or registration reported.	(147)
Isoquercetin	D-gal-induced C28/I2 senescence; ACLT-induced OA in male C57BL/6 mice; paired mildly/severely damaged human knee OA cartilage.	“Mice in the IQ treatment groups received oral administration of IQ daily for 8 weeks	One week post-surgerymice were randomized to shamACLTor ACLT+IQ (100 or 200 mg/kg/day; n=8/group).	IQ reduced modified Mankin scorescartilage degenerationand chondrocyte necrosis in ACLT mice.	IQ pharmacokineticsbioavailabilityand drug interactions remain unassessed; clinical OA evidence is lacking.	Preclinical *in vitro* and *in vivo* evidence	Preclinical OA candidate; no clinical trial results or registration reported.	(148)
Glycoursodeoxycholic acid (GUDCA)	Male mouse DMM/age-related OA models; intestine-specific Fxr-knockout mice and controls; intestinal organoids.	Daily gavage: post-DMMassessed at 8 weeks; aging mice18–24 months of age.	Controlled preclinical study with mechanistic testing (n=7–8/group); human bile acid association cohorts (n=1, 714/154)not GUDCA trials.	GUDCA reduced cartilage damage/osteophytesincreased ACAN/GLP-1and decreased ADAMTS5; benefit was lost with intestinal Fxr deletion or joint GLP-1R blockade.	Male animal models; supplementary dosing data unavailable; human associations and genetic evidence do not establish clinical efficacy.	Preclinical *in vivo*	No GUDCA OA interventional clinical trial reported. OA regulatory approval status not reported	(83)
Curcumin	MIA-induced knee OA in male SD rats; IL-1β-treated primary rat chondrocytes.	*In vivo*: 0.5% curcumin injection for 4 weeks; IV in Methods/**Figure 2** IP in Discussion. Dose/volume and frequency not reported. *In vitro*: 10 μM24 h.	Network pharmacology + randomized animal study (5 groups; n = 10/group) + cell assays. Histology scored by two blinded assessors; cell replicate number not specified.	Reduced OARSI/Mankin scores and MMP13; increased collagen II and mitophagy; improved mitochondrial function. Compound C/CsA attenuated protection.	No human intervention or genetic validation; incomplete dosing details and conflicting route; mitophagy mechanism requires further confirmation.	Preclinical *in vitro* and *in vivo* evidence	Preclinical study; no original clinical trial. OA approval status not reported.	(149)
Vitamin D	MIA-induced OA in male Wistar rats; IL-1β-treated human OA chondrocytes.	Oral vitamin D 100/500 IU/day or 100 IU/day + celecoxib 30 mg/kg/day from post-MIA day 3; behavioral follow-up to day 17. Cells: 20 nM ± celecoxib 1 μM48 h (autophagy: 24 h).	Randomized animal study: n=6/group (Methods)n=4/group (**Figure 1**/5); human cartilage: 5 donorsex vivo.	Reduced paincartilage damage and synovitis; enhanced autophagy. Combination improved some pain/cartilage outcomeswithout added weight-bearing benefit.	Smallshort-term study; inconsistent sample sizesunclear calcitriol/IU dosingand no human efficacy data.	Preclinical *in vitro* and *in vivo* evidence	No original clinical trial; OA approval status not reported.	(150)
Liraglutide (GLP-1R agonist)	MIA-induced knee OA in adult male Wistar rats.	Subcutaneous injection50 μg/kg/day for 28 days.	Two animal experiments: 60 rats/6 groups (time course) and 30 rats/3 groups (treatment); 10/group allocated5/group in figures.	Reduced cartilage TNF-αIL-1β and IL-6; increased GLP-1R and PKA/CREB signals; reduced body weight.	No pain or structural efficacy endpoints; no pathway-blockade validation; weight-loss contribution unresolved; allocation-to-analysis numbers unexplained.	Preclinical *in vivo* evidence	Preclinical study; no human trial. OA approval status not reported.	(151)
Gastrodin	IL-1β-treated primary rat chondrocytes; ACL transection plus medial meniscus resection in male SD rats.	*In vivo*: subcutaneous 100 μg/kg/day after surgery until sacrifice; duration not reported. *In vitro*: key assays 10/50 μM; viability assays 24/48 h.	Randomized animal study: 15 rats3 groupsn = 5/group; cell assays report n = 5 (replicate type unclear).	Reduced apoptosisIL-6/TNF-α and MMP-3; preserved collagen II. Reduced cartilage damage and OARSI scores in rats.	Preclinical *in vitro* and *in vivo* evidence	Small animal sample; treatment duration unclear; no human or pain outcomes; no pathway rescue/genetic validation.	Preclinical study; no human trial. OA approval status not reported.	(152)

## 7 Future research directions and clinical translation

It is worthy of note that in conjunction with the exploration of novel therapeutic targetsdrug repurposing strategies are increasingly manifesting their unique advantages and translational potential in the management of OA (185). Future investigations ought to concentrate on clarifying the metabolic reprogramming of immune cells and resident joint cells including chondrocytes and fibroblast-like synoviocytes as well as their dynamic interaction during the progression of OA (186). This is of pivotal importance for elucidating the core disease mechanisms. For example the M1/M2 polarization state of macrophages is intricately associated with metabolic pathways including glycolysis and fatty acid oxidation. In contrast mitochondrial dysfunction in chondrocytes under OA conditions creates a vicious cycle with the abnormal activation of innate immune pathways such as the cGAS-STING pathway (187). Future research could explore how metabolic interventions can be used to modulate immune cell function while simultaneously investigating how immune modulation may improve the metabolic homeostasis of joint-resident cells (10). Pantethine serves as a relevant case study. Initially utilized for lipid modulation as a coenzyme A precursor recent research has demonstrated its ability to stabilize mitochondrial membranes in bone cells decrease mtDNA leakage and suppress cGAS-STING pathway activation thus effectively alleviating cartilage degradation and synovitis in OA models (54). Similarly statins employed in the treatment of cardiovascular diseasesuch as atorvastatinhave been discovered to mitigate the severity of OA in animal models via mechanisms related to the modulation of cholesterol metabolism and the inhibition of the RhoA/ROCK signaling pathway (185). Furthermore the anti - diabetic agent metformin by virtue of its actions in activating AMPK inhibiting the NF-κB pathway and improving mitochondrial function has shown potential for alleviating inflammation and offering chondro protection in pre-clinical studies of OA. Future research ought to systematically explore the mechanisms of action of repurposed drugs in OA and promote their clinical evaluation (188). Despite the extensive utilization of traditional nonsteroidal anti-inflammatory drugs (NSAIDs)the gastrointestinal and cardiovascular risks associated with long-term administration necessitate careful consideration (189). Nevertheless the optimal target population long-term safety and potential synergistic effects of these agents with metabolic interventions necessitate further investigation (190). By leveraging technologies including metabolomics lipidomics proteomics and single-cell transcript omicsa systematic identification of biomarkers associated with the onset progression patient stratification and therapeutic response of OA can be achieved (191). These biomarkers will not only contribute to the early diagnosis and subtype classification of OA but also assist in guiding therapeutic decision-making. For instance they can be used to determine a patient’s probable response to statinsmetforminor specific biologic agents thereby facilitating the implementation of personalized medicine (192). Future endeavors should encompass the design and implementation of large-scale prospective multi center clinical trials to systematically assess the long-term efficacy and safety of drug repurposing strategies novel metabolic-immune targeted agents and integrated lifestyle interventions in OA. Study designs must appropriately consider patient heterogeneity integrating stratified analyses based on factors such as metabolic syndrome obesity and genetic background (193). Ultimately a multidimensional integrated care model ought to be established. This model should incorporate pharmacotherapy (which includes novel agents and repurposed drugs)nutritional support exercise therapy pain management and patient education. The overarching objective is to comprehensively enhance joint function and the quality of life for patients with OA (194). Continued in-depth clarification of its intricate biological mechanisms will establish the foundation for the discovery of novel therapeutic targets. Meanwhile actively promoting mechanistic research and clinical translation of drug repurposing can provide patients with more rapid and potentially safer therapeutic options (195). By integrating precise biomarkers individualized multimodal interventions and rigorous clinical validationit is possible to fundamentally transform the current OA treatment paradigm shifting from mere symptom control to true disease modification and ultimately enhance the long-term health outcomes and quality of life for patients with OA (196). In the pursuit of therapeutic strategies for OA (197) advanced technologies targeting the immunometabolic axis exhibit significant potential. However their translation from conceptual frameworks to clinical practice continues to be beset with challenges (198). These challenges not only encompass technical hurdles but also involve complexities in clinical application which necessitates in-depth analysis. Firstly attaining tissue-specific targeting is a crucial concern. In diseases such as OA multiple cell types and metabolic pathways are intricately intertwined making mono therapy against a single factor often insufficient (199). The dynamic interaction among immune cells chondrocytes and synoviocytes significantly impacts the inflammatory state and metabolic balance of the joint. As a result the development of targets that consider these complex cellular inter actions along with tissue-specific delivery systems is a prerequisite for precision medicine. Additionally delivery efficiency represents another substantial challenge. Many emerging therapeutic approaches such as gene therapy and engineered cell therapies are restricted by the limitations of their delivery platforms (200). Efficiently guiding therapeutic agents or cells to specific joint compartments and guaranteeing their functionality within the inflammatory microenvironment continues to pose a significant translational challenge. The advancement of nanoparticles microcapsules and other intelligent delivery systems will play a crucial role in resolving this issue. Moreover long - term safety is a vital consideration. The possibility of off-target effects and long-term consequences associated with gene editing or engineered cell therapies demands comprehensive assessment. Overcoming these challenges requires interdisciplinary collaboration to integrate knowledge and resources from multiple fields thus promoting both basic research and clinical translation in OA immunometabolic therapy. Urgently addressing the current obstacles calls for multidisciplinary cooperation to combine diverse expertise and resources facilitating more in - depth research and clinical application of immunometabolic interventions for OA. (**Figure 3**).

**Figure 3:**
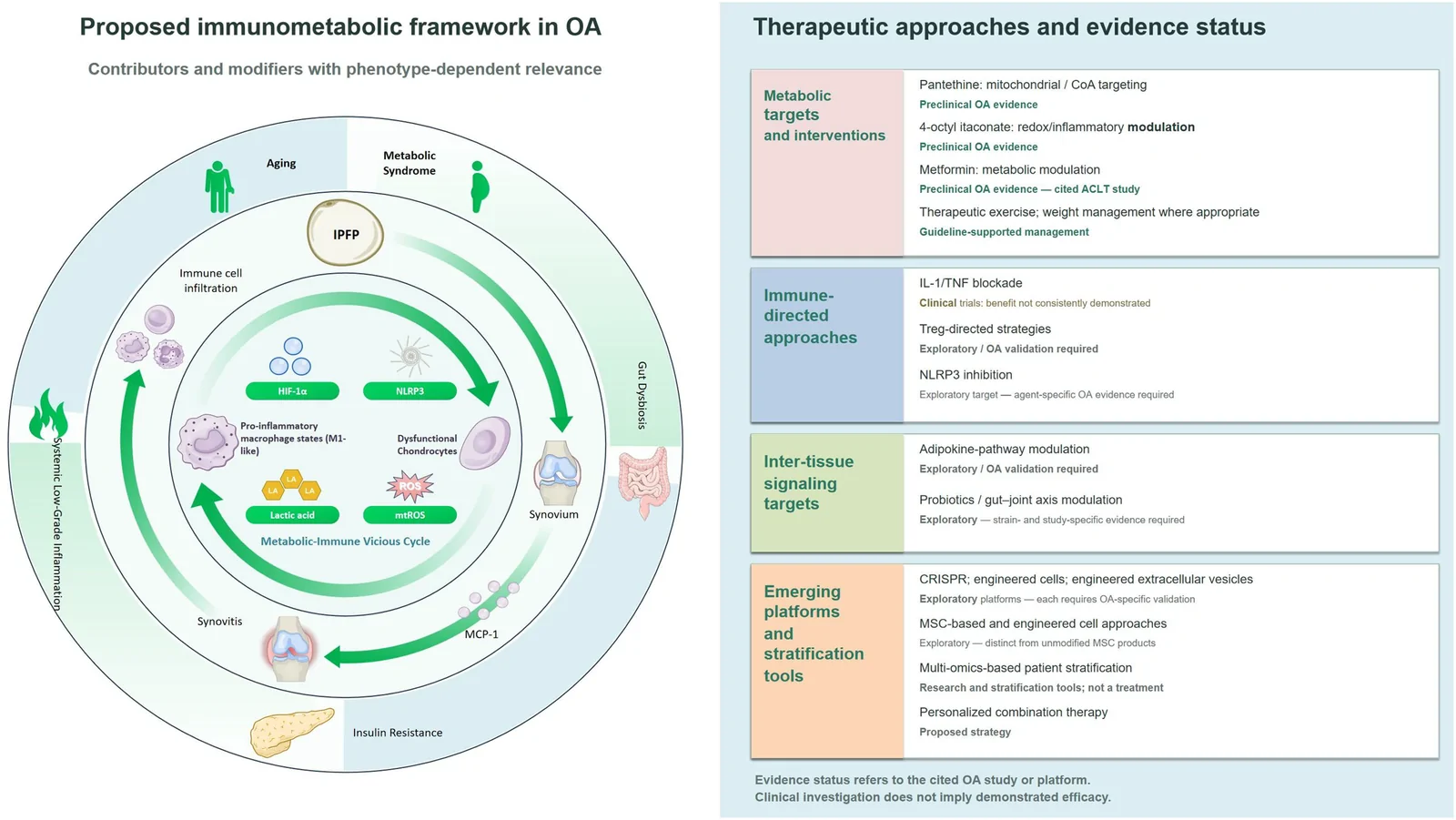
Three-layer pathogenic network of osteoarthritis: metabolic–immune crosstalksystemic driversand treatment strategies. This schematic depicts OA pathogenesis in a simplified three-layer concentric model. At the corea metabolic–immune vicious cycle operates between M1 macrophages and dysfunctional chondrocytes sustained by inflammatory mediators and metabolic byproducts such as HIF-1αNLRP3lactateand mtROSwhich can be targeted by anti-inflammatory agents metabolic modulators antioxidantsor inhibitors of NLRP3 and HIF-1α. In the middle layerthe infrapatellar fat pad (IPFP) amplifies local inflammation through adipokines and FFA spromoting synovitis and immune cell infiltration including MCP-1which can be addressed by IPFP modulation adipokine signaling blockadeor local anti-inflammatory therapies. In the outer layersystemic risk factors and disease modifiers such as aging and metabolic syndrome exacerbate low-grade inflammationin sulin resistance and gut dysbiosisfurther reinforcing intra-articular dysfunction and can be managed through lifestyle interventions metabolic control and gut microbiome modulation.

## 8 Summary

Osteoarthritis (OA) is a heterogeneous whole-joint disease in which mechanical age-related post-traumatic genetic inflammatory and metabolic factors contribute to varying degrees across patients and disease stages. Within this multifactorial framework immunometabolic dysregulation represents a significant pathogenic factor in OA particularly in phenotypes characterized by metabolic abnormalities and persistent low-grade inflammation.Accumulating evidence indicates bidirectional interactions between cellular metabolism and inflammatory signalling in chondrocytes synovial fibroblasts macrophages and other joint-resident or infiltrating cells thereby modulating the progression of OA. In inflammatory OA phenotypes macrophage and T-cell states may contribute to local inflammatory and catabolic signaling through mediators such as TNF-α and IL-1βalthough their relative contributions vary across patients and disease stages. Meanwhile both joint-resident cells and immune cells experience profound metabolic reprogramming. This includes energy crises and oxidative stress resulting from mitochondrial dysfunctionlactic acid accumulation and microenvironment acidification caused by abnormal up-regulation of glycolysis and pro-inflammatory signals generated by lipid metabolism disorders. More importantly metabolic dysregulation provides the material and energy foundation for sustained immune activation while immune inflammatory responses further aggravate cellular metabolic abnormalities thereby forming a self-sustaining vicious cycle at the cellular tissue and systemic levels. Based on this mechanistic comprehension therapeutic strategies are transitioning towards targeting immunometabolic interaction nodes. These strategies involve modulating key pathways such as AMPK/mTOR employing metabolic reprogramming drugs developing biologics targeting specific cytokines or metabolites and integrating advanced biotechnologies such as gene editing synthetic biology and stem cell therapy. Gene editing synthetic biology and engineered cell-based approaches represent emerging experimental strategies for OA. Preclinical studies suggest that these technologies may enable more targeted modulation of inflammatory and regenerative pathways; however their efficacy long-term safety delivery efficiency and clinical applicability remain to be established. Similarly artificial intelligence may facilitate OA risk stratification imaging analysis and clinical decision support but its role in guiding immunometabolic therapy remains investigational and requires prospective clinical validation.
